# Serum myostatin as a candidate disease severity and progression biomarker of spinal muscular atrophy

**DOI:** 10.1093/braincomms/fcae062

**Published:** 2024-02-28

**Authors:** Ana Letícia Amorim de Albuquerque, Júlia Kersting Chadanowicz, Giovanna Câmara Giudicelli, Ana Lucia Portella Staub, Arthur Carpeggiani Weber, Jordana Miranda De Souza Silva, Michele Michelin Becker, Thayne Woycinck Kowalski, Marina Siebert, Jonas Alex Morales Saute

**Affiliations:** Graduate Program in Medicine, Medical Sciences, Federal University of Rio Grande do Sul, Porto Alegre 90035-003, Brazil; Clinical Neurogenetics research group, Hospital de Clínicas de Porto Alegre, Porto Alegre 90035-007, Brazil; Clinical Neurogenetics research group, Hospital de Clínicas de Porto Alegre, Porto Alegre 90035-007, Brazil; Bioinformatics core, Hospital de Clínicas de Porto Alegre, Porto Alegre 90035-007, Brazil; Graduate Program in Genetics and Molecular Biology, Federal University of Rio Grande do Sul, Porto Alegre 91501-970, Brazil; Clinical Neurogenetics research group, Hospital de Clínicas de Porto Alegre, Porto Alegre 90035-007, Brazil; Clinical Neurogenetics research group, Hospital de Clínicas de Porto Alegre, Porto Alegre 90035-007, Brazil; Graduate Program in Medicine, Medical Sciences, Federal University of Rio Grande do Sul, Porto Alegre 90035-003, Brazil; Child Neurology Unit, Hospital de Clínicas de Porto Alegre, Porto Alegre 90035-007, Brazil; Bioinformatics core, Hospital de Clínicas de Porto Alegre, Porto Alegre 90035-007, Brazil; Graduate Program in Genetics and Molecular Biology, Federal University of Rio Grande do Sul, Porto Alegre 91501-970, Brazil; Medical Genetics Service, Hospital de Clínicas de Porto Alegre, Porto Alegre 90035-007, Brazil; Unit of Laboratorial Research, Experimental Research Center, Hospital de Clínicas de Porto Alegre (HCPA), Porto Alegre 90035-007, Brazil; Graduate Program in Gastroenterology and Hepatology, Federal University of Rio Grande do Sul, Porto Alegre 90035-003, Brazil; Graduate Program in Medicine, Medical Sciences, Federal University of Rio Grande do Sul, Porto Alegre 90035-003, Brazil; Clinical Neurogenetics research group, Hospital de Clínicas de Porto Alegre, Porto Alegre 90035-007, Brazil; Medical Genetics Service, Hospital de Clínicas de Porto Alegre, Porto Alegre 90035-007, Brazil; Department of Internal Medicine, Federal University of Rio Grande do Sul, Porto Alegre 90035-003, Brazil

**Keywords:** spinal muscular atrophy, biomarker, myokine, myostatin, follistatin

## Abstract

The identification of biomarkers for spinal muscular atrophy is crucial for predicting disease progression, severity, and response to new disease-modifying therapies. This study aimed to investigate the role of serum levels of myostatin and follistatin as biomarkers for spinal muscular atrophy, considering muscle atrophy secondary to denervation as the main clinical manifestation of the disease. The study evaluated the differential gene expression of myostatin and follistatin in a lesional model of *gastrocnemius* denervation in mice, as well as in a meta-analysis of three datasets in transgenic mice models of spinal muscular atrophy, and in two studies involving humans with spinal muscular atrophy. Subsequently, a case-control study involving 27 spinal muscular atrophy patients and 27 controls was conducted, followed by a 12-month cohort study with 25 spinal muscular atrophy cases. Serum levels of myostatin and follistatin were analysed using enzyme-linked immunosorbent assay at a single centre in southern Brazil. Skeletal muscle gene expression of myostatin decreased and of follistatin increased following lesional muscle denervation in mice, consistent with findings in the spinal muscular atrophy transgenic mice meta-analysis and in the *iliopsoas* muscle of five patients with spinal muscular atrophy type 1. Median serum myostatin levels were significantly lower in spinal muscular atrophy patients (98 pg/mL; 5–157) compared to controls (412 pg/mL; 299–730) (*P* < 0.001). Lower myostatin levels were associated with greater disease severity based on clinician-rated outcomes (Rho = 0.493–0.812; *P* < 0.05). After 12 months, there was a further reduction in myostatin levels among spinal muscular atrophy cases (*P* = 0.021). Follistatin levels did not differ between cases and controls, and no significant changes were observed over time. The follistatin:myostatin ratio was significantly increased in spinal muscular atrophy subjects and inversely correlated with motor severity. Serum myostatin levels show promise as a novel biomarker for evaluating the severity and progression of spinal muscular atrophy. The decrease in myostatin levels and the subsequent favourable environment for muscle growth may be attributed to denervation caused by motor neuron dysfunction.

## Teaser

De Albuquerque *et al*. report the potential of serum myostatin and follistatin levels as biomarkers for spinal muscular atrophy, encompassing research with animal models and humans. Myostatin levels were reduced in the disease, correlated with a disease severity and progressed over time, suggesting its utility as disease severity and progression biomarker.

## Graphical Abstract

**Graphical Abstract:**
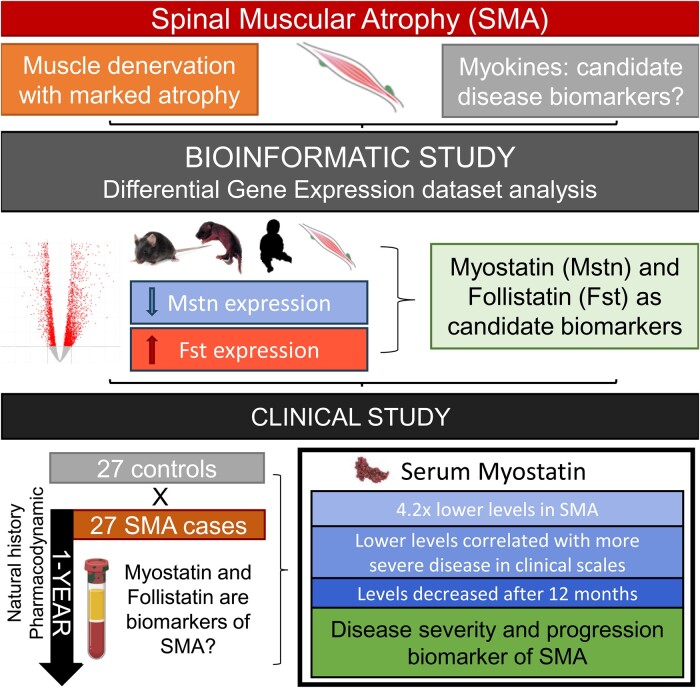

## Introduction

Spinal muscular atrophy (SMA) is an autosomal recessive neurodegenerative disease characterized by progressive motor neuron degeneration.^1-3^ Its incidence is estimated at 1 in 6000–10 000 live births, with a prevalence of 1 in 40–60 individuals as mutation carriers in the general population.^4,5^ SMA is a leading cause of infant mortality and the second most common fatal autosomal recessive disease.^1-3^

The disease is caused by bi-allelic pathogenic variants in the survival of motor neuron 1 gene (*SMN1*), resulting in the absence of SMN protein production.^1-3,6^ The complete absence of this protein would be lethal during the embryonic period, however, the presence of the *SMN2* gene, a paralogous gene that varies in copy number, allows for the production of a small amount of functional SMN protein.^1-3^ The severity of SMA exhibits significant variation, and its clinical manifestations can be classified into five sub-types.^1^ Type 0 is the most severe form, occurring in the neonatal period. Type 1 is characterized by flaccid paralysis and the inability to sit without support, with symptoms emerging before 6 months of age. Type 2 has an onset between 6 and 18 months, where children can achieve independent sitting but are unable to walk independently. Type 3 is a more heterogeneous form, affecting children or young individuals who can walk independently but gradually experience limb weakness progression. Type 4 is the mildest form, with disease onset occurring after 18 years of age.^1-3^ Advancements in understanding SMA pathophysiology have led to significant progress in disease-modifying therapies targeting SMN levels like nusinersen, onasemnogene abeparvovec and risdiplam.^3,7,8^ Early initiation of therapy has shown the greatest effectiveness, particularly in pre-symptomatic children with 2–3 copies of *SMN2*^7,8^. However, individual patients’ clinical courses and disease progression sometimes deviate from the typical natural history, suggesting variations in treatment response regardless of age at initiation.^9,10^

Therefore, it is important to identify SMA biomarkers for assessing disease severity, progression, prognosis, and therapeutic response. Previous studies have examined blood markers such as *SMN* mRNA and protein levels,^11^ leukocyte DNA methylation profiles,^11,12^ and plasma proteins identified through unbiased proteomic studies.^13^ However, these markers lacked clear differentiation among SMA sub-types and showed limited association with disease severity, reducing their clinical utility.^13^ The plasma levels of neurofilament heavy chain (pNF-H)^14,15^ and light chain (NfL)^16-19^ have demonstrated potential as biomarkers for axonal damage, reflecting disease severity and treatment response in infantile-onset SMA. However, these findings have not been reproducible in patients with SMA types 2 and 3, nor in chronic stages of SMA.^17-19^

Considering SMA as a neuronal disease with clinical manifestations primarily occurring due to denervation-induced skeletal muscle alterations, we aimed to evaluate the potential of myostatin, a negative regulator of muscle growth, its antagonist, follistatin, and its ratio as biomarkers. We analysed skeletal muscle differential gene expression (DGE) data from lesional denervation and transgenic mice models of SMA, as well as from humans with SMA in a bioinformatic study. Subsequently, we assessed the serum levels of myostatin and follistatin in a clinical study to investigate their potential as biomarkers for disease severity, progression, and treatment response to novel therapies.

## Materials and methods

### Study design

The study consisted of two phases. The first phase involved a bioinformatic study in which the analysis of candidate myokine expression was performed using raw data from DGE studies in muscle or blood samples from humans and mice, obtained from public repositories. The second phase included a cross-sectional case-control study, followed by a cohort study conducted at a single centre.

### Bioinformatic study

To investigate the potential alteration of myostatin and follistatin in SMA due to muscle denervation, we initially manually selected a study that examined the temporal DGE of myokines in the *gastrocnemius* muscle of mice following tibial nerve denervation.^20^ After that, transcriptome datasets evaluating SMA disease were searched on publicly available repositories Gene Expression Omnibus (GEO)^21^ and ArrayExpress (AE).^22^ The following terms were used as keywords:*‘(SMA) OR (SMA AND muscle) OR (SMA AND blood) OR (SMA) OR (SMA AND muscle) OR (SMA AND blood)’.* Filters were applied to meet the inclusion criteria of studies conducted on blood or muscle samples of humans and/or mice through microarray and/or RNA-Seq methodologies. Experiments performed in cell lines, with less than three samples per group, without control groups, or without raw data available in the databases were excluded.

### Clinical study

The first phase of the clinical study involved a cross-sectional case-control study, where serum levels of myostatin and follistatin were measured in both groups. Additionally, in the case group, clinician-rated outcomes (ClinRO) were assessed: Children’s Hospital of Philadelphia Infant-Test-of-Neuromuscular-Disorders (CHOP-INTEND); Hammersmith-Infant Neurological-Examination (HINE); Hammersmith-Functional-Motor-Scale-Expanded (HFMSE); and Revised-Upper-Limb-Module (RULM). SMA functional status was categorized as non-sitters; sitters (defined as able to sit without support, stand alone or stand with assistance); and walkers (defined as able to walk independently). In the second phase, a longitudinal cohort was conducted exclusively with the case group. This cohort involved a 12-month follow-up period during which the analysis of biomarkers was repeated.

Subjects in the study were recruited consecutively from May to October 2021 at the Neuromuscular Genetics outpatient clinic of Hospital de Clínicas de Porto Alegre and included individuals with a confirmed genetic diagnosis of SMA-5q type 1, 2 or 3 undergoing regular follow-up.

The control group consisted of healthy participants with a body mass index (BMI) appropriate for their age, selected based on sex and age matching with the case group. The control group was recruited from the hospital community through invitations extended to healthy relatives of patients consulting at the hospital due to other diseases or patients seeking consultation for other health issues that did not meet exclusion criteria (e.g. those on the waiting list for umbilical or inguinal hernia surgery, etc.). Individuals with other neurological or systemic conditions that could lead to additional motor impairments or negatively impact cardiopulmonary performance, such as stroke sequelae, chronic obstructive pulmonary disease, decompensated heart failure, severe asthma, were excluded from both groups.

The study was conducted in accordance with ethical guidelines and was approved by the institutional research ethics committee (GPPG-HCPA-2020-0731). Informed consent was obtained from all participants, adhering to the ethical principles outlined in the Declaration of Helsinki.

### Serum analysis

Three mL of serum samples were obtained from peripheral venous blood of participants and controls and placed in serum separator tubes. The samples were left at room temperature (18–25°C) for 30–45 minutes to clot, then centrifuged at 1000 × *g* for 10 min at a temperature of 23°C. Care was taken to avoid haemolysis and the separated serum was stored in a freezer at −80°C until analysis. Serum levels of myostatin and follistatin were measured in duplicate with a commercially available enzyme-linked immunosorbent-assay kit (R&D-Systems, DuoSet^®^ Growth/differentiation factor 8/Myostatin, cat. no. DY788-05, and DuoSet^®^ human Follistatin, cat. no. DY669, Minneapolis, MN, USA), in accordance with the manufacturer’s instructions.

### Statistical analysis

#### Datasets processing and meta-analysis

The microarray studies were manually extracted through GEO/AE and later analysed with the *affy* package^23^ in R v4.2.2^24^ and RStudio v2022.7.2.576 softwares applying a robust multiarray average (RMA) normalization. The raw data from the RNA-Seq datasets were processed on the useGalaxy server^25^ as follows: .fastq quality was evaluated in the FastQC,^26^ alignment was performed using the Bowtie2,^27^ and quantification using the featurecounts^28^ tools. DGE analysis was conducted for each dataset in R and RStudio using the *limma* package^29^ for the microarray studies and *edgeR* package^30^ for the RNA-Seq assays. Principal Component Analysis was performed for each dataset to evaluate the heterogeneity of the samples. The surrogate variable analysis normalization was performed to reduce batch effects. The resulting genes from the DGE analysis with logFC ≥ 1 and *P*-value ≤ 0.05 were considered upregulated, whilst genes with logFC ≤ -1 and *P*-value ≤ 0.05 were considered downregulated.

#### Analysis of the clinical study

Statistical tests were selected according to the distribution of data given by Shapiro–Wilk test and histograms. The variables in the study did not have normal distribution and were shown as median (interquartile ranges), except for BMI, CHOP-INTEND, HFMSE and RULM which are presented as mean (95% confidence intervals, CI). Comparisons between paired cases and controls were performed with Wilcoxon signed-rank test, with corrections for weight and BMI (covariates) performed using rank analysis of covariance (Quade’s test), or Chi-Square. Comparisons among SMA sub-types, functional status and *SMN2* copy numbers were performed with Kruskal–Wallis test. Comparisons between SMA treated and untreated subjects at baseline were performed with Mann–Whitney U-test. Changes in myostatin and follistatin serum levels and their ratio from baseline to 12 months of follow-up were performed with Wilcoxon signed-rank test. Correlations between myokines levels at baseline with age, age at onset, reported disease duration and the ClinROs were performed with Spearman correlation-test. We also performed Spearman correlation-test searching for the association of the difference in myokines levels from 12 months to baseline (DeltaMyokine) with disease severity variables at baseline and time since onset of treatment (TSOT). TSOT considered the number of days of treatment when baseline samples were collected, with positive values indicating subjects already taking the treatment at baseline, zero meaning that treatment was started at baseline visit and negative values that treatment was started after baseline. Untreated patients were censored for this last analysis.

## Results

### Bioinformatic study: transcriptome datasets

#### Temporal pattern of myostatin and follistatin DGE in muscle submitted to lesional denervation

**Figure 1:**
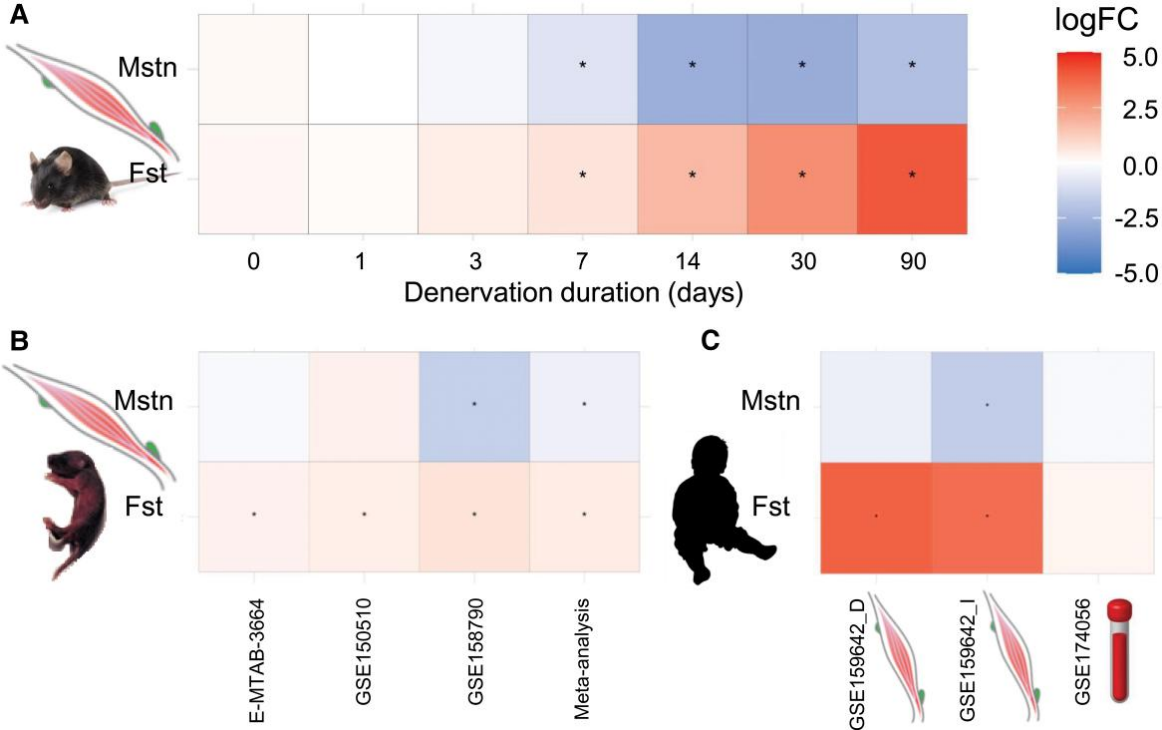
**Heat-map of the differential expression of myokines in acute and chronic neurogenic skeletal muscle atrophy in mice.** (**A**) DGE of *Mstn* and *Fst* RNA in *gastrocnemii* of C57BL/6J male mice after 0 (baseline), 1, 3, 7, 14, 30 or 90 days of tibial nerve denervation from Ehmsen *et al.*, 2019 dataset. Significant gastrocnemius atrophy is induced within one week after denervation in this model, with atrophy becoming progressively more severe over time. (**B**) DGE of *Mstn* and *Fst* RNA in two studies with the Taiwanese model Smn–/–; *SMN2* (E-MTAB-3664 and GSE150510, with a similar proportion of male and female mice) and one with the SMN Delta7 model (GSE158790, sex of mice not informed) and the meta-analysis of the three studies. (**C**) DGE of *MSTN* and *FST* RNA in post-mortem diaphragm and iliopsoas samples from SMA patients (GSE159642) and on whole-blood samples of SMA type 1 patients and controls (GSE174056). *indicates statistical significance with *P*-values. Heat colours indicate increased while cold colours indicate reduced expression of RNA when compared to control tissue. DGE analysis was conducted for each dataset in R and RStudio using the edgeR. DGE analysis with logFC ≥ 1 and *P*-value ≤ 0.05 were considered upregulated, whilst genes with logFC ≤ −1 and *P*-value ≤ 0.05 were considered downregulated. D, diaphragm; I, *iliopsoas*; LogFC, log-fold changes.

To investigate the potential alterations in myokine regulation due to muscle denervation and atrophy in SMA, we examined the DGE of myostatin (*Mstn*) and follistatin (*Fst*) in a mouse model of neurogenic skeletal muscle atrophy.^20^ We analysed RNA-seq data from the *gastrocnemius* muscle of male mice following lesional denervation of the tibial nerve. The time points evaluated included baseline, 1, 3, 7, 14, 30 and 90 days post-lesion, with four mice per group. Our analysis revealed a significant decrease in *Mstn* expression and an increase in *Fst* expression in the denervated muscle, starting at 7 days post-lesion and persisting until 90 days (Fig. 1A, Supplementary Table 1 in Supplementary material).

#### Systematic review of public databases on DGE in SMA

The systematic review of GEO/AE databases identified three relevant studies with RNA-Seq in mice, two conducted with the Taiwanese model Smn–/–; *SMN2* SMA mice.^31-33^ one with the SMN Delta7 model.^29^ In two studies, DGE was assessed in the *tibialis anterior* muscle, with one study examining postnatal day 7 in three transgenic mice and three controls of both sexes (GSE150510)^33^ and the other examining postnatal day 10 in four transgenic mice (sex not informed) and five controls (GSE158790).^32^ The third study (E-MTAB-3664)^31^ evaluated skeletal muscle in four affected and four control mice (similar proportion of male and females) at postnatal days 1 and 4,^31^ which were grouped together before analysis. A decrease in *Mstn* expression in SMA mice was found on the GSE158790 dataset and the meta-analysis of all three studies. Conversely, *Fst* expression was found to be increased in SMA mice across all three datasets and in the meta-analysis (Fig. 1B, Supplementary Table 1 in Supplementary material).

We identified two human studies that assessed transcriptomes in muscle tissue or blood samples. In the study conducted by Ramos and colleagues^34^ (GSE159642), DGE was examined in post-mortem diaphragm and *iliopsoas* samples from five patients with SMA type 1 (aged 4–16 months, except for one case with unknown age) and one additional diaphragm sample from a patient with SMA type 2 (72 months old). These samples were compared to three control samples from individuals who died of unknown causes/cardiac arrest (aged 19–168 months). The findings of this study demonstrated a similar pattern of DGE to the mice studies, with decreased expression of *MSTN* and increased expression of *FST* in the *iliopsoas* (I) muscle of SMA patients, as well as increased expression of *FST* in the diaphragm (D) (Fig. 1C, Supplementary Table 1 in Supplementary material). In the study GSE174056, DGE analysis was performed on whole-blood samples using RNA-seq. This study included five patients with SMA type 1 (all with two copies of *SMN2*) under 1 year of age (121–259 days) and five healthy age-matched controls under 1 year old.^35^ The results showed no significant differences in the expression of myostatin (LogFC = −0.1894; *P*-value = 0.891) or follistatin (LogFC = 0.2449; *P*-value = 0.757) (Fig. 1C). Due to the limited number of eligible human studies, a meta-analysis could not be performed.

These results supported the hypothesis that myostatin and follistatin could serve as potential biomarkers for SMA, warranting further evaluation in a clinical study.

### Clinical study

A total of 54 participants were included in the study, comprising 27 homozygous cases with the common deletion in the *SMN1* and 27 healthy individuals. The detailed clinical, genetic, and demographic characteristics of the participants can be found in Table 1. At baseline, 11 out of 27 (40.7%) cases were receiving disease-modifying therapies related to SMN levels. All of them received nusinersen, and four out of the eleven (27.3%) cases switched to onasemnogene abeparvovec. During the 12-month follow-up period, four subjects initiated treatment and one patient transitioned from nusinersen to onasemnogene abeparvovec.

**Table 1:** Main demographics, clinical and genetic characteristics of the study sample at baseline

Variable	Controls (*n* = 27)	SMA subjects (*n* = 27)
Male	14 (51.8%)	13 (48.2%)
Age—mo	143 (40–252)	132 (38–276)
Age at onset—mo	–	7 (2–12.5)
Disease duration—mo	–	110.5 (32.25–252.75)
Weight—kg	53.65 (15.87–75.75)	35 (14.54–60)
BMI—kg/m²	20.7 (19.01–22.38)	19.93 (16.85–23.0)
SMA type		Type 1–12/27 (44.4%)
	–	Type 2- 9/27 (33.3%)
		Type 3–6/27 (22.2%)
*SMN2* copy number		2 copies—13/27 (48.1%)
	–	3 copies—10/27 (37%)
		4 copies—3/27 (11.1%)
*Functional status*	Walkers 27/27 (100%)	Non-sitters 10/27 (37%) Sitters 15/27 (55.5%) Walkers 2/27 (7.4%)
*Ventilatory support*	0/27 (0%)	13 (48.1%)
CHOP-INTEND *(N = 17)*	–	25.29 (16.16–34.43)
HINE (*N* = 16)	–	4.25 (1.32–7.18)
HFMSE (*N* = 8)	–	19.5 (8.57–30.43)
RULM (*N* = 6)	–	22.83 (12.12–33.5)
On disease-modifying therapies	–	11 (40.7%)
Use of nusinersen	–	11 (40.7%)
Use of Onasemnogene abeparvovec	–	4 (14.8%)

Data are shown as median (interquartile range) or frequencies (percentages), except for BMI which is shown as mean (95% confidence interval). BMI, body mass index; CHOP INTEND, Children’s Hospital of Philadelphia Infant Test of Neuromuscular Disorders; HINE, Hammersmith Infant Neurological Examination; HFMSE, Hammersmith Functional Motor Scale–Expanded; Mo, months; RULM, revised upper limb module; SMA, spinal muscular atrophy.

#### Serum levels of myokines

##### Myostatin

**Figure 2:**
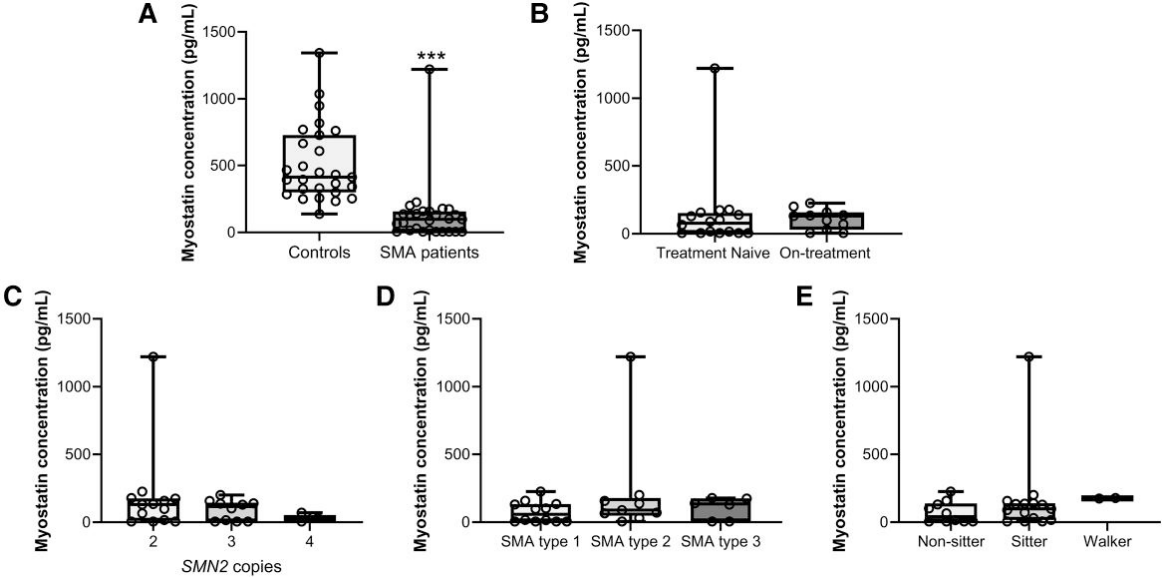
**Myostatin serum levels at baseline.** SMA, spinal muscular atrophy. ***P < 0.001. Paired comparisons between cases and controls (**A**) were performed with Wilcoxon signed-rank test, where *Z* = −4.036 and *P* < 0.001. Comparisons between SMA treated, and untreated subjects at baseline were performed with Mann–Whitney U-test (**B**), comparisons among *SMN2* copy numbers (**C**), SMA sub-types (**D**), and functional status (**E**) were performed with Kruskal–Wallis test. Dots represent patient’s single data points.

Serum myostatin levels were significantly lower in subjects with SMA, with a median of 98 pg/mL (5–157 pg/mL), compared to controls who had a median of 412 pg/mL (299–730 pg/mL; *P* < 0.001 corrected for weight or BMI; Fig. 2A). Median myostatin levels were 40.92 pg/mL (5–137.52 pg/mL) in the non-sitters group, 97.85 pg/mL (17.42–139.8 pg/mL) in the sitters group, and 176.2 pg/mL (174.4-ND pg/mL) in the walkers group (*P* = 0.138, Fig. 2E). There was no significant difference in myostatin levels among SMA sub-types (*P* = 0.477, Fig. 2B), based on *SMN2* copy number (*P* = 0.465, Fig. 2C), treatment status (*P* = 0.422, Fig. 2D), or gender (*P* = 0.830).

**Figure 3:**
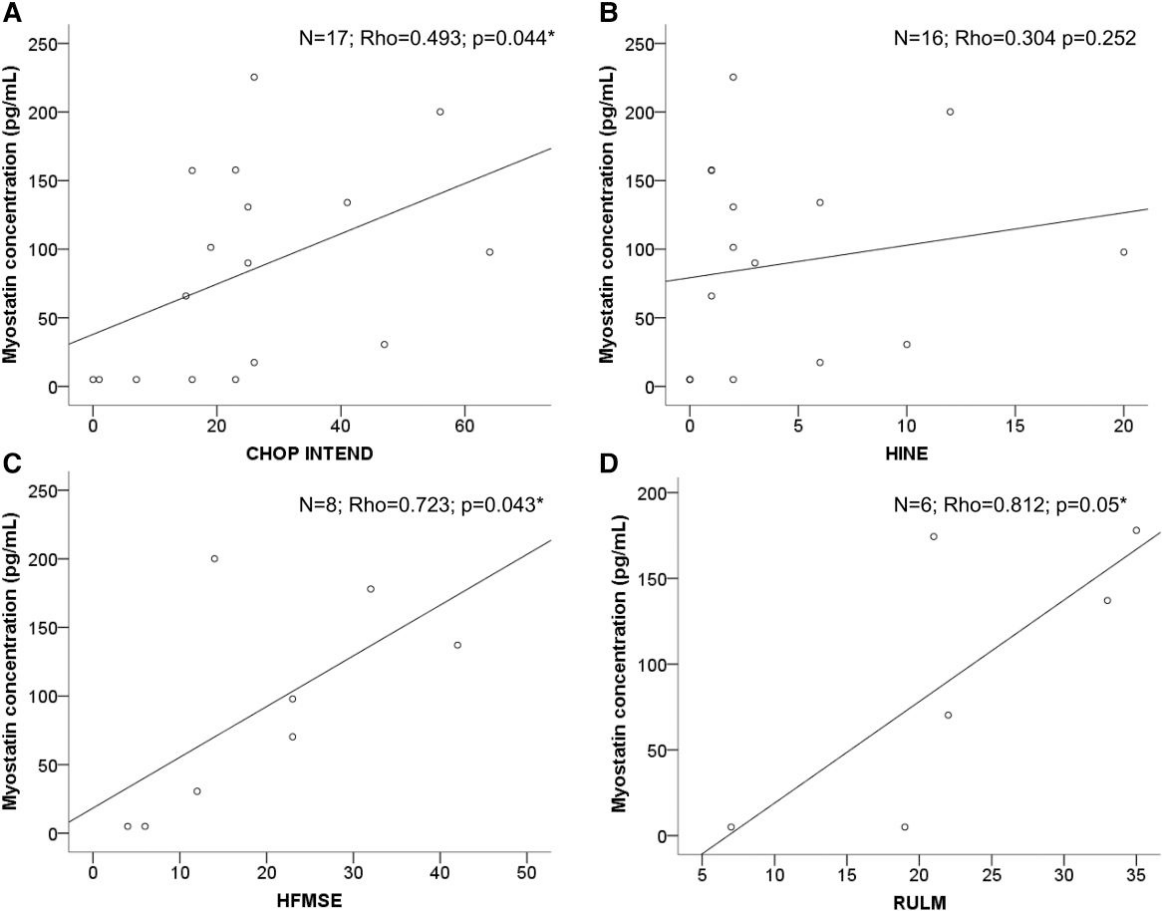
**Serum myostatin levels correlation with ClinRO.** Myostatin levels correlation with CHOP INTEND are depicted in (**A**), with HINE in (**B**), with HFMSE in (**C**) and with RULM in (**D**). CHOP INTEND, Children’s Hospital of Philadelphia Infant Test of Neuromuscular Disorders; HINE, Hammersmith Infant Neurological Examination; HFMSE, Hammersmith Functional Motor Scale–Expanded; RULM, Revised Upper Limb Module. *P < 0.05. Dots represent patient’s single data points.

There was moderate to strong direct correlations between myostatin levels and CHOP-INTEND (N = 17; Rho = 0.493, *P* = 0.044, Fig. 3A), HFMSE (*N* = 8; Rho = 0.723, *P* = 0.043, Fig. 3C) and RULM scores (*N* = 6; Rho = 812, *P* = 0.05, Fig. 3D), in which lower myostatin levels were associated with worse motor performance (lower scale scores). However, there was no significant correlation between serum myostatin levels and HINE (*N* = 16; Rho = 0.304, *P* = 0.252, Fig. 3B). No significant correlations were found between serum myostatin levels with age (Rho = 0.041, *P* = 0.840), weight (Rho = 0.023, *P* = 0.910), BMI (Rho = 0.167, *P* = 0.414), age at onset of symptoms (Rho = 0.185, *P* = 0.365), or disease duration (Rho = −0.004, *P* = 0.986).

**Figure 4:**
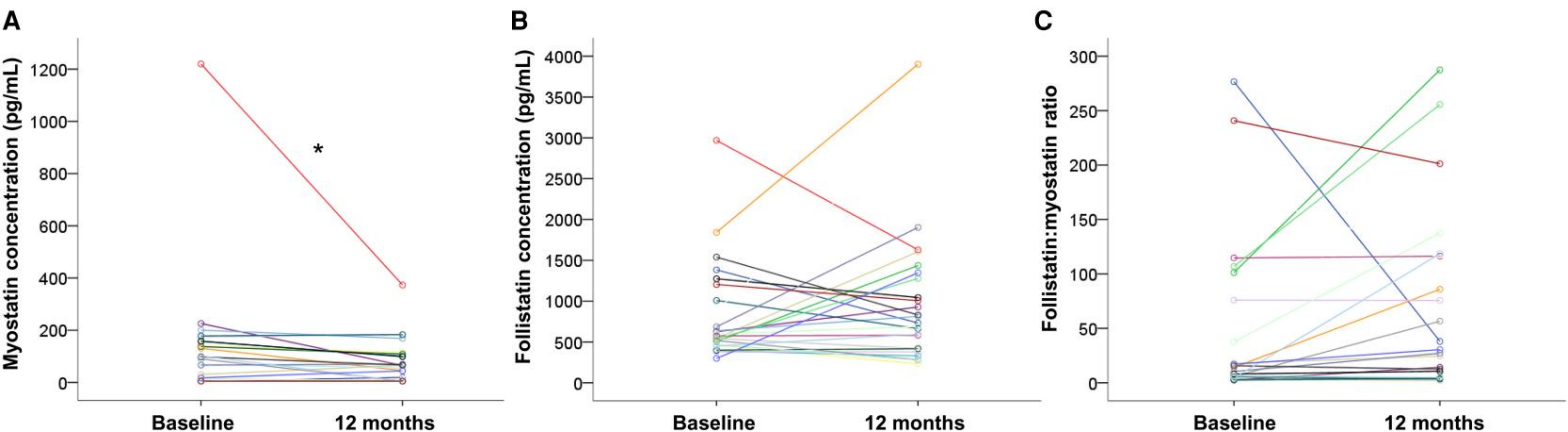
**Myokines levels on longitudinal 12-months follow-up.** SMA, spinal muscular atrophy. *P < 0.05. Paired comparisons were performed with Wilcoxon signed-rank test, were *Z* = −2.427 and *P* = 0.015 for myostatin levels analysis (**A**). Progression of follistatin levels is depicted in (**B**) and of follistatin:myostatin ratio in (**C**). Dots represent patient’s single data points.

During the 12 months of follow-up, there was evidence of an additional reduction in myostatin levels to 55 (5–104.5) pg/mL (*P* = 0.021, Fig. 4A). However, there was no correlation between the change in myostatin levels over time and the severity of the disease at baseline (*P* > 0.05 for all comparisons) or the TSOT variable (Rho = 0.022, *P* = 0.940).

##### Follistatin

There was no significant difference in serum follistatin levels between SMA cases and controls (cases: 573.5 pg/mL, controls: 790.8 pg/mL, *P* = 0.288—*P* = 0.438 corrected for weight and *P* = 0.760 corrected for BMI, Supplementary Fig. 1A in Supplementary material). Similarly, no differences were observed in follistatin levels among SMA sub-types (*P* = 0.610, Supplementary Fig. 1B in Supplementary material), based on *SMN2* copy number (*P* = 0.109, Supplementary Fig. 1C in Supplementary material), between treated and untreated patients (*P* = 0.272, Supplementary Fig. 1D in Supplementary material), among functional status groups (*P* = 0.680, Supplementary Fig. 1E in Supplementary material) or between genders (*P* = 0.458).

Follistatin levels only showed a correlation with age at symptoms onset (Rho = −0.425, *P* = 0.030), while no significant correlations were found with age (Rho = −0.208, *P* = 0.297), weight (Rho = −0.244, *P* = 0.219), BMI (Rho = −0.300, *P* = 0.137), disease duration (Rho = −0.192, *P* = 0.349), HINE (Rho = 0.113, *P* = 0.676), CHOP-INTEND (Rho = 0.279, *P* = 0.278), HFMSE (Rho = 0.084, *P* = 0.844), or RULM (Rho = −0.429, *P* = 0.397).

There was no significant change in follistatin levels over the 12-month follow-up period (*P* = 0.493), with levels measuring 813.0 pg/mL (418.25–1392.0 pg/mL) at the end of the study (Fig. 4B). Additionally, there was no correlation between the changes in follistatin levels during the study and the baseline disease severity variables or the TSOT variable (*P* > 0.05 for all comparisons).

##### Follistatin:myostatin ratio

**Figure 5:**
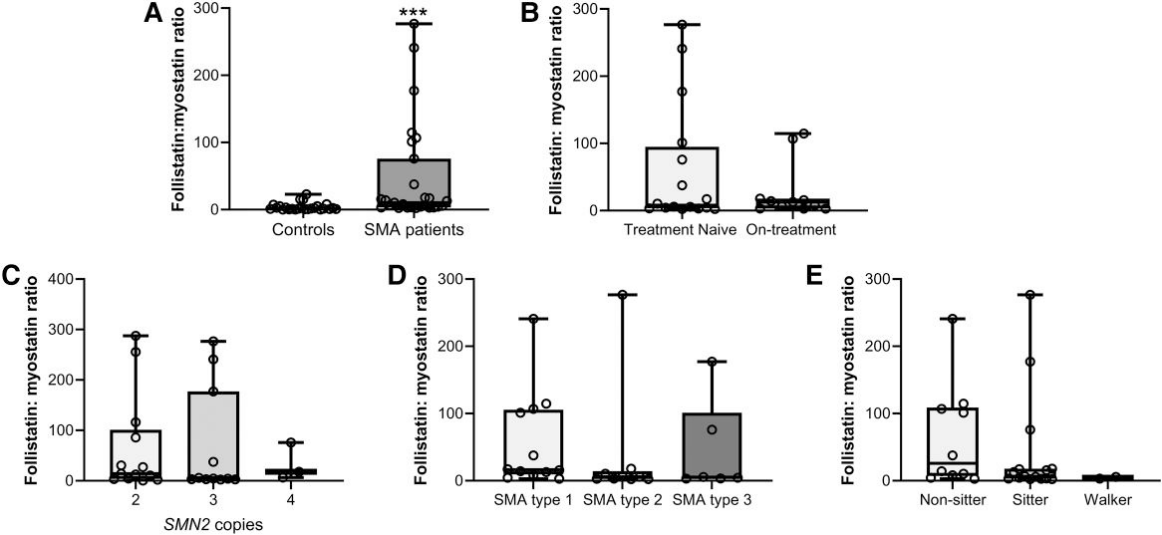
**Follistatin:myostatin ratio at baseline.** ***P < 0.001. Paired comparisons between cases and controls were performed with Wilcoxon signed-rank test, *Z* = 3.646 and *P* < 0.001 for follistatin:myostatin ratio analysis (**A**). Comparisons between SMA treated, and untreated subjects at baseline were performed with Mann–Whitney U-test (**B**), and comparisons among SMN2 copy numbers (**C**), SMA sub-types (**D**), and functional status (**E**) were performed with Kruskal–Wallis test. Dots represent patient’s single data points.

The follistatin:myostatin ratio was significantly increased in SMA subjects (10.39; 3.28–75.9) compared to controls (1.55; 0.73–5.15, *P* < 0.001 corrected for weight or BMI, Fig. 5A). However, there was no difference in the follistatin:myostatin ratio between different SMA sub-types (*P* = 0.160, Fig. 5B), based on the number of *SMN2* copies (*P* = 0.790, Fig. 5C), between treated and untreated patients (*P* = 0.942, Fig. 5D), among functional status groups (*P* = 0.215, Fig. 5E) or between genders (*P* = 0.905, data not shown).

The follistatin:myostatin ratio showed a significant inverse correlation with HFMSE scores (N = 8; Rho = −0.850, *P* = 0.007, Supplementary Fig. 2C in Supplementary material). Although not statistically significant, similar trends were observed for CHOP-INTEND (Supplementary Fig. 2A in Supplementary material), HINE (Supplementary Fig. 2B in Supplementary material), and RULM (Supplementary Fig. 2D in Supplementary material), where higher ratios were associated with lower scores indicative of greater disease severity. The follistatin:myostatin ratio did not show significant correlations with age (Rho = −0.188, *P* = 0.348), weight (Rho = −0.170, *P* = 0.396), BMI (Rho = −0.297, *P* = 0.141), age at onset of symptoms (Rho = −0.295, *P* = 0.144), or disease duration (Rho = −0.115, *P* = 0.575). There was a non-statistically significant trend towards an additional increase in the follistatin:myostatin ratio over the 12-month follow-up period (*P* = 0.083), with a ratio of 27.13 (4.59–115.5) at the end of the study (Fig. 4C). The difference in the follistatin:myostatin ratio during the study correlated only with disease duration at baseline (Rho = −0.404, *P* = 0.045), while no significant correlations were found with other disease severity variables at baseline or with the TSOT variable (*P* > 0.05 for all other comparisons).

## Discussion

Gene expression analysis in transgenic SMA models, as well as in chronically denervated mice muscle and muscle tissue samples from SMA patients in our bioinformatic study supported a potential role for myostatin and follistatin as biomarkers for SMA. The subsequent clinical study confirmed the significance of myostatin as a biomarker, as SMA patients exhibited a substantial reduction in serum levels. Notably, individuals with more severe motor impairments demonstrated even lower myostatin levels, which further declined after a 12-month follow-up period. Conversely, serum follistatin levels did not differ significantly between SMA cases and controls, resulting in an increased follistatin:myostatin ratio in SMA. The decreased gene expression of myostatin over time in acutely denervated skeletal muscle tissue along with an increased follistatin:myostatin ratio, mirrored the observations in SMA patients, suggesting that the denervation process resulting from motor neuron dysfunction/death in SMA may be a key component responsible for lower myostatin serum levels.

### Myostatin

Myostatin, a member of the transforming growth-factor ß (TGF-ß) family, is primarily expressed and secreted by skeletal muscle and serves as a negative regulator of muscle growth. It exerts its effects by binding to plasma membrane-associated activin type IIB and type IIA (ActRIIB/IIA) receptors, leading to inhibition of muscle stem cell proliferation and fusion, as well as modulation of local protein synthesis.^36,37^ Previous studies have demonstrated reduced myostatin levels in various muscular diseases, including muscular dystrophies, myotonic disorders, and inclusion body myopathies.^38,39^ In a previous study, serum myostatin levels were assessed in four patients with infantile-onset SMA, with a mean age of 11 years at assessment, demonstrating a significant reduction compared to controls and even lower levels than those in patients with primary myopathies.^40^ Our findings not only confirm this anecdotal report but also establish a correlation between lower myostatin levels and greater motor disease severity according to ClinROs, indicating its potential as a biomarker of disease severity. Additionally, we observed a progressive reduction in myostatin levels over time, suggesting its role as a candidate biomarker of disease progression. Of note, a potential floor effect was identified for myostatin serum levels in advanced disease stages. Seven patients with SMA had myostatin levels at the detection threshold of the method, with four out of the seven being patients with SMA type 1 aged between 3 and 8 years, all in advanced stages, and one out of the seven being a patient with SMA type 3 with a disease duration of 38 years. In all of these cases, myostatin levels at the 12-month follow-up remained at the detection threshold of the method, including in a SMA type 1 patient for whom treatment with nusinersen was initiated on the same day as baseline sample collection.

The results of the clinical study were reinforced by the DGE analyses, which revealed decreased myostatin expression in denervated *gastrocnemius* muscle in mice, skeletal muscle of SMA transgenic mice, and *iliopsoas* muscle (severely affected muscle) of SMA patients with advanced disease. The unaltered gene expression of myostatin in whole blood of SMA patients^35^ was expected, considering its preferential expression in muscle tissue.^37^ Reduced *Mstn* expression started after 7 days of tibial nerve lesion, which is coincident with the onset of muscle atrophy in that model.^20^ However, taking into account the consistent findings of reduced gene expression of *Mstn* in muscle tissue when normalized by protein levels across the transgenic mice and human study in SMA, it is more likely that altered regulation of myostatin, rather than muscle loss alone, explains the reduced circulating myostatin levels in SMA, similar to what is considered for other neuromuscular diseases.^41^ The lack of correlation between myostatin levels and weight and BMI further supports this interpretation; however, future studies correcting myostatin serum levels for lean mass based on dual energy X-ray absorptiometry or MRI scans and also assessing its muscle tissue expression in a larger sample of SMA subjects in different functional status and sub-types will be important to better characterize the source of reduced myostatin levels in SMA. The progressive reduction in myostatin expression following acute tibial nerve injury indicates that denervation processes play a crucial role in the downregulation of myostatin. Thus, it is plausible to suggest that in SMA denervation resulting from dysfunction or death of motor neurons in the anterior horn of the spinal cord is a major contributing factor to the altered regulation of myostatin.

No differences in myostatin levels were observed between patients treated with disease-modifying therapies and drug-naïve individuals, nor was there an association between the annual variation in myostatin levels and the variable TSOT. It is important to note that the present study was not designed or adequately powered to detect differences in candidate treatment response biomarkers. The inclusion of 11 patients undergoing treatment at the baseline assessment, as well as four patients who started treatment on the same day or after sample collection, may have introduced a confounding factor when assessing the differences in biomarkers compared to controls and in relation to disease severity and SMA sub-types. The sample of patients included in the treatment group was biased towards individuals with early and more severe forms of the disease, as 83.3% of SMA type 1 patients and 55.5% of SMA type 2 patients were receiving treatment. Conversely, the untreated group consisted mostly of individuals with late-onset forms of the disease, as no patients with SMA type 3 were under treatment. Considering the significant correlation between lower myostatin levels and more severe disease based on ClinROs, it is conceivable to speculate that treatment could have led to increased myostatin levels in the early and severe forms of the disease, acting as a confounding factor in the comparative analysis among SMA sub-types and in relation to the number of *SMN2* copies. Additionally, studies conducted in the SMN Delta7 mouse model have demonstrated that neonatal treatment with antisense oligomers to restore SMN expression can increase myostatin expression in muscle tissue, approaching levels similar to those in controls, thus highlighting the potential of myostatin as a treatment response biomarker for novel disease-modifying therapies.^42^ Similarly, an interesting study in the Golden Retriever muscular dystrophy model showed the role of myostatin as a treatment response biomarker in other neuromuscular diseases.^38^ Of note, the confounding factor related to access to disease-modifying treatments in different SMA sub-types would play a diminished role in comparing myokine levels between groups based on functionality. For instance, patients with SMA type 1 responding to treatment could exhibit a similar functional status to patients with SMA type 2 (sitters). We observed lower levels of myostatin in the non-sitters group, followed by the sitters group, with the highest values observed among cases in the walkers group. However, given that the magnitudes of the differences were smaller compared to those observed between cases and controls, the study lacked the statistical power to detect such distinctions. Future studies with larger sample sizes will be essential to assess differences in myostatin levels among SMA functional statuses. Additionally, investigations exclusively involving treatment-naïve patients will aid in evaluating differences among sub-types and their correlation with the number of SMN2 copies. Furthermore, studies focusing primarily on evaluating myostatin as a treatment response biomarker for novel disease-modifying therapies, with larger sample sizes stratified by different SMA sub-types, and including patients ranging from early to advanced disease stages are warranted.

### Follistatin

Follistatin, a single-chain glycoprotein, is expressed in most tissues where activin mRNAs are present. It functions by inhibiting the binding of myostatin and other members of the TGF-ß family to ActRIIB/IIA receptors, playing a crucial role in muscle fibre formation, growth, and hypertrophy.^43,44^ In our study, serum levels of follistatin were slightly elevated compared to controls, which is consistent with the previous anecdotal study involving four SMA cases,^40^ but the difference was not statistically significant. We did not observe any correlation between follistatin levels and other clinical indicators of disease severity or disease progression. These results differ from gene expression studies that consistently showed increased follistatin expression in the skeletal muscle of transgenic SMA mice, SMA patients, and denervated mouse *gastrocnemius* muscle. Unlike myostatin, follistatin exhibits a broader expression profile, with liver expression being >8 times greater than skeletal muscle expression.^45^ Consequently, increased expression of follistatin in muscle may not necessarily result in elevated serum levels of this myokine. Furthermore, changes in gene expression do not necessarily lead to alterations in protein levels, as there are many post-transcriptional regulatory mechanisms at play. In this sense, the role of serum follistatin as a biomarker for neuromuscular diseases is less clear compared to myostatin, with some studies reporting normal levels,^46,47^ while others indicate increased levels^40,37^ compared to controls.

### Follistatin:myostatin ratio

The increase in the follistatin:myostatin ratio appears to be a consequence of the reduction in myostatin levels in SMA and is associated with positive regulators of muscle growth in healthy individuals, promoting muscle hypertrophy.^48^ While not directly demonstrated, it can be inferred that the study involving four cases of SMA also showed an increase in the follistatin:myostatin ratio, considering the slightly elevated levels of follistatin and the reduced levels of myostatin observed.^40^ These findings differ from a study conducted in amyotrophic lateral sclerosis, where a decrease in the follistatin:myostatin ratio was observed in patients compared to controls.^42^ Similarly, a recent study in a sub-type of limb-girdle muscular dystrophy reported similar results to our study, with an increased follistatin:myostatin ratio that correlated with disease severity variables. In both studies, correlations were stronger when myostatin was evaluated independently rather than in the context of the follistatin:myostatin ratio.^47^

### Therapeutic implications

Several studies in animal models of SMA have investigated the inhibition of myostatin as a potential therapeutic target, often utilizing follistatin or follistatin analogues.^40,43,49-53^ Initial studies using recombinant follistatin administration in the SMN Delta7 mouse model demonstrated increased muscle mass, improved motor performance, and extended survival, regardless of SMN levels. However, more recent studies in the same model using transgenic inactivation of myostatin or transgenic overexpression of follistatin did not replicate these results.^41^ More promising results, albeit with smaller effect sizes, have been observed in animal models of SMA with milder disease phenotypes.^41^ Our study’s findings, showing a marked reduction in myostatin levels in SMA and increased follistatin:myostatin ratio indicating a favourable environment for muscle growth, raise questions regarding the biological plausibility of myostatin inhibition as a highly effective therapeutic strategy for the disease. However, this does not contradict the potential combined use of disease-modifying therapies targeting SMN alongside myostatin pathway inhibition. In this regard, a recent study demonstrated a synergistic effect of these interventions in SMN Delta7 mice.^41,42^ In that study, myostatin expression was restored to levels similar to controls using SMN-related therapy, and within this context, myostatin inhibition led to increased body weight, muscle mass, fibre size, motor function, and physical performance.

### Study limitations

One of the limitations of our study is that it is a single-centre study with a relatively small sample size. As we only found one study that measured myostatin levels in four patients with infantile-onset SMA and long-term illness, we conducted an exploratory study considering the differences between cases and controls in serum myostatin and follistatin levels and their ratio, all as main outcomes, without statistical corrections for multiple comparisons. Despite the limitations of our convenience sample, we were able to identify significant differences in myostatin levels and the follistatin:myostatin ratio. Regarding follistatin levels, we conducted a sample size calculation using the final data, considering an 80% power to detect a type II error and a 5% significance level. The calculation indicated that the sample size would need to be increased to 506 subjects, which is not feasible for a study on a rare disease. Therefore, it was considered that the study had sufficient power to identify relevant differences in the outcomes of interest. The similarity of our results with those of the anecdotal study^40^ and the congruence with the findings of the DGE studies for myostatin indicate the robustness of the data. The heterogeneity of the SMA population, encompassing different SMA sub-types, and the recruitment during COVID-19 pandemic, resulted in a limited number of patients with complete clinical rating outcomes (ClinROs) available. It is crucial for future studies with larger sample sizes to assess the correlations between myostatin levels and ClinROs more comprehensively among SMA sub-types. Another limitation is the lack of power to assess the potential of serum myostatin levels as a treatment response biomarker. Future longitudinal studies are necessary to address this question and to compare the sensitivity of myostatin serum levels to changes in ClinROs and to determine if baseline myostatin levels and their changes over time can predict treatment response. Finally, it will be important to compare the biomarker properties (disease severity, progression and pharmacodynamic) of myostatin with that of other simpler to evaluate and widely available muscle markers like serum creatinine and creatine kinase and of pNF-H and NfL levels in future studies.

## Conclusion

In conclusion, our study highlights myostatin as a potential biomarker for disease severity and progression in SMA, with its reduction likely linked to the denervation process caused by dysfunction or loss of anterior horn neurons. The findings also suggest that targeting myostatin alone may not be an effective therapeutic strategy for SMA, given the already substantial inhibition of this pathway in the disease. Instead, combining myostatin inhibition with SMN-related therapies may hold promise. Future research should focus on investigating the role of myostatin as a treatment response biomarker in the context of disease-modifying therapies for SMA.

## Supplementary material

Supplementary material is available at *Brain Communications* online.

## Supplementary Material

fcae062_Supplementary_Data

## Data Availability

Data not provided in the article because of space limitations may be shared (anonymized) at the request of any qualified investigator for purposes of replicating procedures and results.
