# Supplementary material for: Serum myostatin as a candidate disease severity and progression biomarker of spinal muscular atrophy
Source: Brain Commun. 2024 Feb 28;6(2):fcae062. doi: 10.1093/braincomms/fcae062 (PMC10939446; doi:10.1093/braincomms/fcae062)
Supplement: fcae062_Supplementary_Data [file fcae062_supplementary_data.pdf]

Supplementary Material

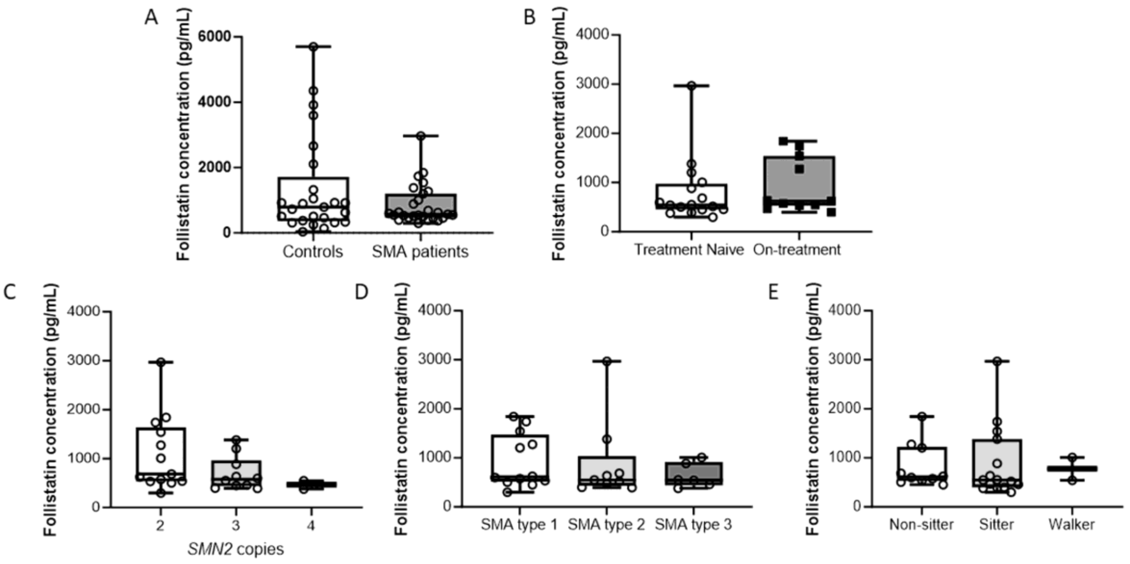

Supplementary Figure 1 - Follistatin serum levels at baseline

SMA, spinal muscular atrophy. Paired comparisons between cases and controls were performed with Wilcoxon signed-rank test, comparisons among SMA subtypes, SMN2 copy numbers and functional status were performed with Kruskal-Wallis test, comparisons between SMA treated, and untreated subjects at baseline were performed with Mann-Whitney U-test. Dots represent patient´s single data points.

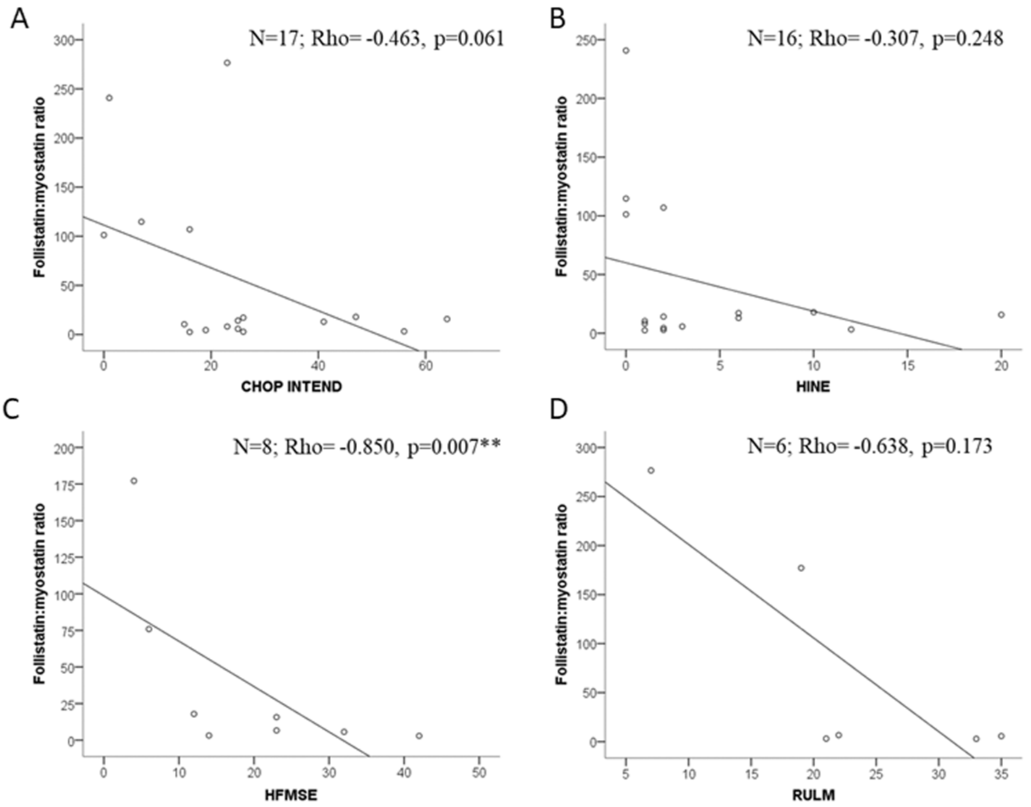

Supplementary Figure 2 – Follistatin:myostatin correlation with clinician-rated outcomes

CHOP INTEND, Children's Hospital of Philadelphia Infant Test of Neuromuscular Disorders; HINE, Hammersmith Infant Neurological Examination; HFMSE, Hammersmith Functional Motor Scale–Expanded; RULM, Revised Upper Limb Module. **$p<0.01$.

Supplementary Table 1 - Raw Data for Figure 1 Construction

**Figure 1A**			
**Gene**	**logFC**	**PValue**	**TimePoint**
Fst	0,191408806	0,408906701	T0
Mstn	0,139965283	0,592381572	T0
Fst	0,091166729	0,754277118	T1
Mstn	-0,01319385	0,96701727	T1
Fst	0,438985913	0,138883995	T3
Mstn	-0,26008919	0,434308684	T3
Fst	0,685841843	**0,000131166**	T7
Mstn	-0,86605756	**2,63E-05**	T7
Fst	1,798596852	**3,86E-24**	T14
Mstn	-2,74536811	**4,41E-25**	T14
Fst	2,854563495	**1,12E-43**	T30
Mstn	-2,79792807	**9,10E-28**	T30
Fst	4,068657227	**7,44E-67**	T90
Mstn	-2,15519595	**3,59E-15**	T90
**Figure 1B**			
**Gene**	**logFC**	**PValue**	**Study**
Fst	0,358511011	**0,023240438**	EMTAB3664
Mstn	-0,17273535	0,599122296	EMTAB3664
Fst	0,424315073	**0,020061818**	GSE150510
Mstn	0,380877797	0,093235257	GSE150510
Fst	0,703142214	**9,02E-05**	GSE158790
Mstn	-1,61260483	**2,23E-15**	GSE158790
Fst	0,495322766	**0,010681987**	Metanalysis
Mstn	-0,46815413	**4,15E-11**	Metanalysis
**Figure 1C**			
**Gene**	**logFC**	**PValue**	**Study**
Fst	3,860960786	**1,23E-02**	GSE159642_D
Mstn	-0,51174608	0,718116217	GSE159642_D
Fst	3,6011036	**2,50E-10**	GSE159642_I
Mstn	-1,67776447	**0,040879732**	GSE159642_I
Fst	0,244949141	0,756560427	GSE174056
Mstn	-0,18939162	0,891339902	GSE174056
